# Suppression of GLI sensitizes medulloblastoma cells to mitochondria-mediated apoptosis

**DOI:** 10.1007/s00432-016-2241-1

**Published:** 2016-09-06

**Authors:** Zhongxiao Lin, Sisi Li, Hansong Sheng, Ming Cai, Lin Yuan Si Ma, Liuxun Hu, Shangyu Xu, Li Sheng Yu, Nu Zhang

**Affiliations:** 1 Department of Neurosurgery, The Second Affiliated Hospital of Wenzhou Medical University, 109 Xueyuanxi Road, Wenzhou, 325000 Zhejiang People’s Republic of China; 2 Department of Rehabilitation Medicine, The Second Affiliated Hospital of Wenzhou Medical University, Wenzhou, 325000 Zhejiang People’s Republic of China

**Keywords:** Medulloblastoma, SHH signalling pathway, GANT61, GLI, Bcl-2, Mitochondria-mediated apoptosis

## Abstract

**Purpose:**

The sonic hedgehog (SHH) signalling pathway plays the important role in medulloblastoma (MB). Altered GLI expression plays a key role in these processes, and the inhibition of GLI may be a good cancer-targeted therapy. This study aimed to investigate whether GANT61, a GLI inhibitor, may inhibit the SHH signalling pathway promoting cell mitochondria-mediated apoptosis and enhance cisplatin apoptosis antineoplastic therapy.

**Methods:**

In our study, we determined the effect of GANT61-mediated inhibition of GLI in Daoy MB cells. Cells were treated with different concentrations of GANT61 alone or in combination with cisplatin. Cell proliferation was assessed with CCK-8 assays, and cell invasion and migration were performed using 8-µm transwell inserts. Cell apoptosis was assessed with flow cytometric analysis and rhodamine 123. qPCR was used to complete RNA experiments. Protein expression was assessed with Western blotting.

**Results:**

The GANT61 significantly inhibited cell proliferation. GANT61 decreased the cell migration and invasion, impairing these crucial steps in tumour progression. Cell apoptosis was significantly increased in Daoy cells. Rhodamine 123 assay showed that GANT61 could decrease the mitochondrial membrane potential promoting cell mitochondria-mediated apoptosis. GANT61 inhibited the expression of GLI and Bcl-2 at both the mRNA and protein levels and might affect the expression of Bax, caspase-3 and caspase-9 to promote cell intrinsic apoptosis. Furthermore, GANT61 could enhance cisplatin-induced apoptosis to decrease the IC50 value of cisplatin. Finally, data suggest that GANT61 could enhance cisplatin-induced apoptosis through promoting the expression of Bax, caspase-3 and caspase-9 protein levels.

**Conclusion:**

Our data suggest that the SHH signalling pathway plays an important role in MB. GLI is an oncogenic transcription factor in the SHH pathway, and targeting GLI with GANT61 results in favourable antitumour activity and targeted therapy.

## Introduction

Medulloblastoma (MB) is one of the most aggressive brain tumours in the paediatric patient and is characterized by high mortality and poor prognosis (Gerber et al. 2014). Through current multimodality therapy, many patients have good prognosis; however, many of them suffer from considerable disability and morbidity (Moxon-Emre et al. 2014; Rutkowski et al. 2010). Therefore, it is necessary to increase understanding of the pathogenesis underlying MB to develop satisfied targeted therapy.

The sonic hedgehog (SHH) signalling pathway has an important role in embryonic development and in the regulation of a variety of cellular functions (Scales and de Sauvage 2009). Aberrant activation of SHH signalling has been implicated in several human cancers, including MB, basal cell carcinoma and pancreatic cancer (Ozgur et al. 2015; Von Hoff et al. 2009). The zinc finger transcription factor GLI, including GLI1 and GLI2, is considered a mediator of the SHH pathway in MB. GLI is possibly an oncogenic gene and is also involved in cell proliferation and apoptosis. To shed light on the role of GLI in MB, we screened for genes preferentially regulated by GLI in MB cells.

GLI has also been implicated in the mitochondrial apoptosis signalling pathways; for example, the apoptosis genes caspase-3 and caspase-9 were identified as direct transcriptional targets of GLI (Graab et al. 2015). The role of GLI in the induction of apoptosis has also been investigated in the several human cancer cells expressing Bcl-2 (Wang et al. 2010). We show here that expression of the typical oncogene Bcl-2 and Bax is predominantly activated by GLI. Bcl-2 plays an important role in the intrinsic apoptotic pathway, acting as the protection of the mitochondria by inhibiting the activation of Bax, caspase-3 and caspase-9 (Marsden et al. 2002, 2004). Caspases are crucial mediators of programmed cell death. Among them, caspase-3 is an important activated death protease and is essential for certain processes associated with apoptosis. Many research report that, in a wide variety of tumour cells, GANT61-induced apoptosis is also dependent on the expression of caspases (Fu et al. 2013; Matsumoto et al. 2014a; Mazumdar et al. 2011a, b; Pan et al. 2012a; Wickstrom et al. 2013). Therefore, the overexpression of Bax, caspase-3 and caspase-9 in conjunction with the inhibition of GLI activity might suppress Bcl-2 activation, accelerating apoptosis.

Many research reports that, in several tumour cell types, the forced overexpression of Bcl-2 has been considered resistance to chemotherapeutic agents (Pilco-Ferreto and Calaf 2016; Wang and Teng 2016). Chemotherapeutic drugs have important roles in anticancer therapy, because they can induce DNA double-strand breaks, accelerating to tumour cells apoptosis. But tumour cells often generated resistant to chemotherapeutic drugs, it is very important to determine the path mechanisms of the drug resistance. Many reports have showed that GLI protein level decreases in a dose-dependent manner in response to GANT61 treatment (Kramann et al. 2015; Moshai et al. 2014; Srivastava et al. 2014). GLI is considered to be a master regulator of the SHH pathway by regulating the genes expression that are crucial for cell apoptosis. GLI is overexpression in several human cancers, indicating that targeting GLI could be an attractive therapeutic strategy against human cancers (Desch et al. 2010; Pan et al. 2012b; Stecca and Ruiz 2010). GANT61 is one of the agents identified as having inhibitory effects on the SHH pathway (Desch et al. 2010; Mazumdar et al. 2011a, b; Mechlin et al. 2010). It acts by selectively binding to GLI and has also been found to suppress tumour proliferation (Mechlin et al. 2010). In our study, GANT61 had in vitro activity against tumour proliferation and induced cell apoptosis. Furthermore, we found that GANT61 can inhibit the expression of GLI mRNA and protein. Suppressing the expression of GLI1 inhibited the overexpression of Bcl-2 and the proliferation of tumour cells while simultaneously promoting cell apoptosis.

In summary, these researches show that GANT61 is an effective treatment for MB. In our study, we determined the antitumour effects and the apoptosis mechanisms of GANT61. We further determined the ability of GANT61 to sensitize Daoy cells to cisplatin, which is commonly used to treat MB. Thus, it may be an attractive therapeutic strategy to block the expression of GLI in MB.

## Materials and methods

### Reagents and antibodies

GANT61 purchased from Sigma-Aldrich (St. Louis, MO, USA) was dissolved in DMSO and stored at −20 °C. Cisplatin was obtained from Sigma-Aldrich and was dissolved at a stock concentration of 2 mmol/L. Rhodamine 123 was purchased from Sigma-Aldrich. Foetal bovine serum (FBS) and 0.25 % trypsin/EDTA were bought from Gibco Life Technologies (Carlsbad, CA, USA). A FITC-annexin V kit was purchased from Abcam (Cambridge, MA, USA). The CCK-8 cell count kit for cell proliferation analysis was purchased from Tongren Chemical Research Institute (Kyushu, Japan). The reverse transcription kit was purchased from Takara (Shiga, Japan). SYBR Green I was purchased from Noble Ryder (Beijing, China). Antibodies for GLI1 (ab49314), GLI2 (ab26056), Bax (ab10813), BCL-2 (ab59348), caspase-3 (ab32351) and caspase-9 (ab25758) were purchased from Abcam (Cambridge, MA, USA). The β-actin antibody (AP0060) was purchased from Bioworld (Louis Park, MN, USA).

### Cell culture

The Daoy MB cell line was purchased from ATCC. Daoy cells were maintained in RPMI 1640 medium supplemented with 10 % foetal bovine serum at 37 °C with 5 % CO_2_. Before each experiment, trypan blue staining was used to define the cell viability. The cell viability was determined to be over 98 %.

### Cell proliferation analysis

The cell proliferation assay was assessed with CCK-8. Daoy cells in the exponential growth phase were pipetted into single cells after trypsin digestion. Eight thousand cells were seeded in each well of a 96-well plate. RPMI 1640 medium containing 10 % FBS was used to culture the cells for 24 h before being replaced by serum-free medium. The cells were starved for 6 h and then incubated in RPMI 1640 medium containing 1 % FBS. Different concentrations of GANT61 (10, 20 and 40 μM) were added with or without cisplatin, with normally growing cells as the negative control and cell-free wells as the blank control. Each group was set with six replicates. The cells were continually cultured in the incubator for another 24 h before the culture medium was removed. One hundred microlitres of fresh RPMI 1640 medium and 10 μL of CCK-8 solution were then added into each well. Cytotoxicity was also assessed with CCK-8 assays. Cells were cultured in the different concentrations of cisplatin (0–70 µM/L) with or without GANT61 (10 µM/L) for 24 h. The cells were placed in the incubator to avoid light. Absorbance at 450 nm was measured at 30 min, 1, 2 and 4 h. The proliferation inhibition rate was calculated as follows: (A450 of negative control group−A450 of GANT61-treated group)/A450 of negative control group × 100 %.

### Cell invasion and migration assay

Cells were treated with the different concentrations of GANT61 (10, 20 and 40 μM) for 24 h, and equal numbers of cells were treated with serum-free medium. Each group was assessed with migration assays or invasion assays. The mean number of cells was calculated in five randomly selected high-power fields.

### Flow cytometry

Cells were collected into 10-mL centrifuge tubes and centrifuged for 5 min at 500–1000 rpm. The culture medium was discarded. Cells were then washed once with the incubation buffer and centrifuged for 5 min at 500–1000 rpm. PI was dissolved at a final concentration of 1 μg/mL in the incubation buffer along with FITC-annexin V to generate the marking buffer. Resuspended cells were labelled with 100 μL of solution buffer in the dark for 10–15 min at room temperature. Cells were then precipitated by centrifugation at 500–1000 rpm for 5 min and washed with incubation buffer. The sample was incubated at 4 °C for 20 min in the dark without shaking. For the flow cytometry analysis, the excitation and detection wavelengths for the FITC channel were 488 and 515 nm, respectively, while another filter with a wavelength greater than 560 nm was used for PI.

### Rhodamine 123

Daoy cells were seeded on glass coverslips and treated with different concentrations of GANT61. Twenty-four hours after incubation, the cells were fixed with rhodamine 123 for 30 min. Then, the cells were washed with PBS. The cells were mounted and observed under a fluorescence microscope.

### PCR array analysis

Cells were treated with RPMI 1640 medium plus 10 % FBS. The medium was changed to RPMI 1640 medium plus 10 % FBS without (control) or with GANT61 after 24 h. Total RNA was extracted from the cells with TRIzol. Then it was treated with PrimeScript™ RT Master Mix (Takara, Japan) for removal of contaminating DNA. Then, samples were tested with a PCR array (Takara, Japan). Data were analysed with the ΔΔCt method.

### Western blotting

Cells were synchronized in RPMI 1640 medium plus 10 % FBS and exposed to different concentrations of GANT61 for 24 h. We measured the protein profile of the cells with Western blot analysis. Cells were collected and washed with PBS three times. Then, the cells were lysed in fresh RIPA protein lysis buffer containing 1 % PMSF on ice. The total protein concentration was determined with the BCA method. After separation by SDS–PAGE electrophoresis, the samples were transferred to PVDF membrane. Protein blots were visualized with Ponceau S staining. The membrane was then blocked for 2 h at room temperature with gentle shaking. GLI1, GLI2, Bax, BCL-2, caspase-3 and caspase-9 antibodies were added for an overnight incubation at 4 °C. The membrane was then incubated with the secondary antibody (1:10,000) at room temperature for 1 h and washed three times with TBST buffer. ECL chemiluminescence reagent and Bio-Rad exposure apparatus were used for exposure.

### Statistical analysis

The statistical software SPSS 19.0 was used for statistical analysis. Data were statistically analysed using analysis of variance. IC50 for each drug was calculated from linear transformation of dose–response curves. All experimental data are expressed as the mean ± standard deviation (SD). *P < 0.05 indicates a statistically significant difference.

## Results

### GANT61 inhibits the cell migration and invasion

**Fig. 1:**
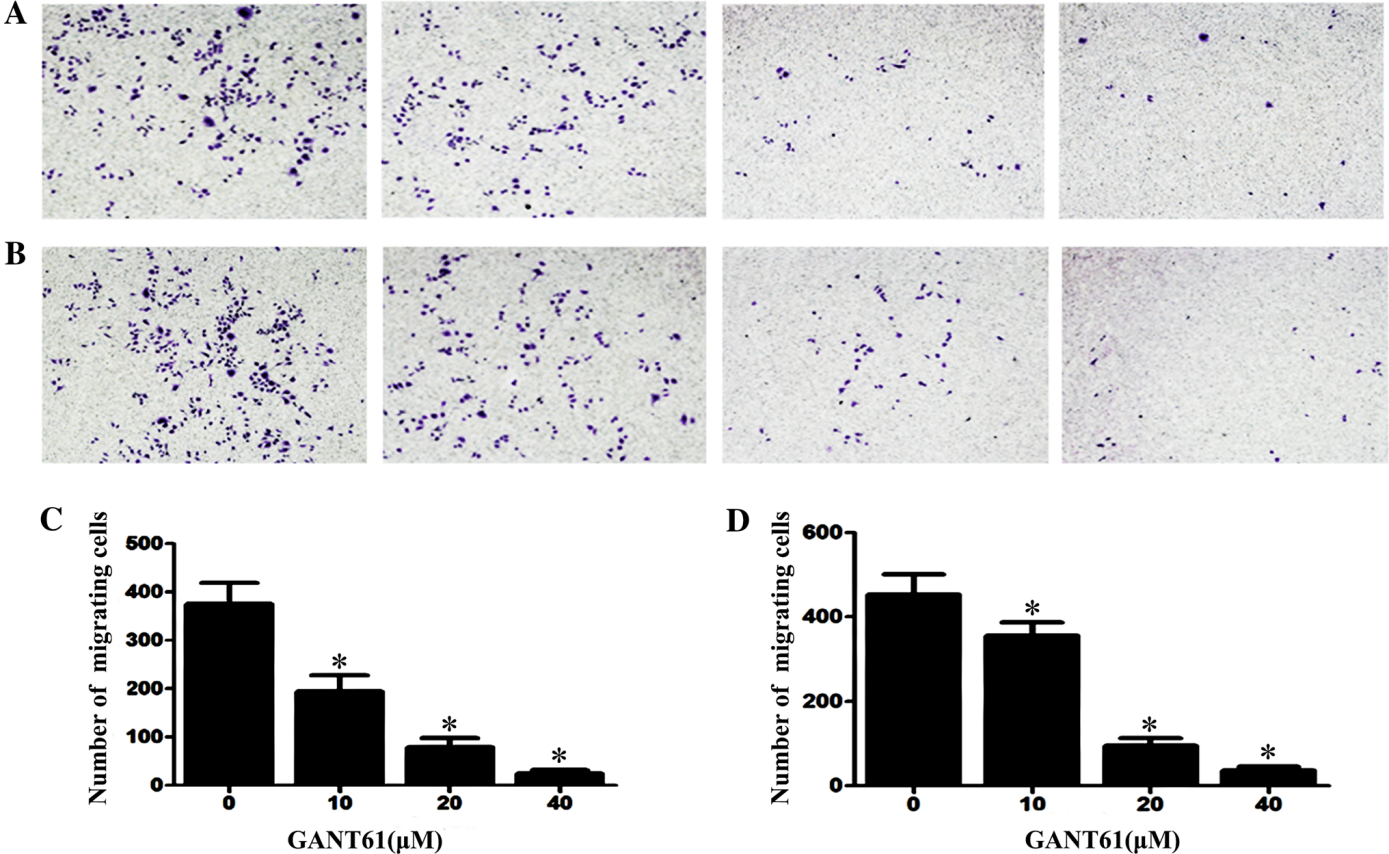
GANT61 *inhibits* the cell migration and invasion. **a**, **b** The migration and invasion capacity was assessed with transwell assays. **c**, **d** *GANT61* inhibited the cell migration and invasion capacity in a dose-dependent manner (*P* < 0.05). The cells were subjected to inverted microscopic observation (×100)

GLI plays an important role in tumour metastasis. Because tumour cell migration and invasion are important steps in tumour metastasis, we determined the effects of *GANT61* on cell migration and invasion. As shown in Fig. 1a, b, the migration and invasion capacity was assessed with transwell assays. Figure 1c, d shows that GANT61 inhibited the cell migration and invasion capacity in a dose-dependent manner (*P* < 0.05).

### GANT61 inhibits proliferation and promotes apoptosis of Daoy cells

**Fig. 2:**
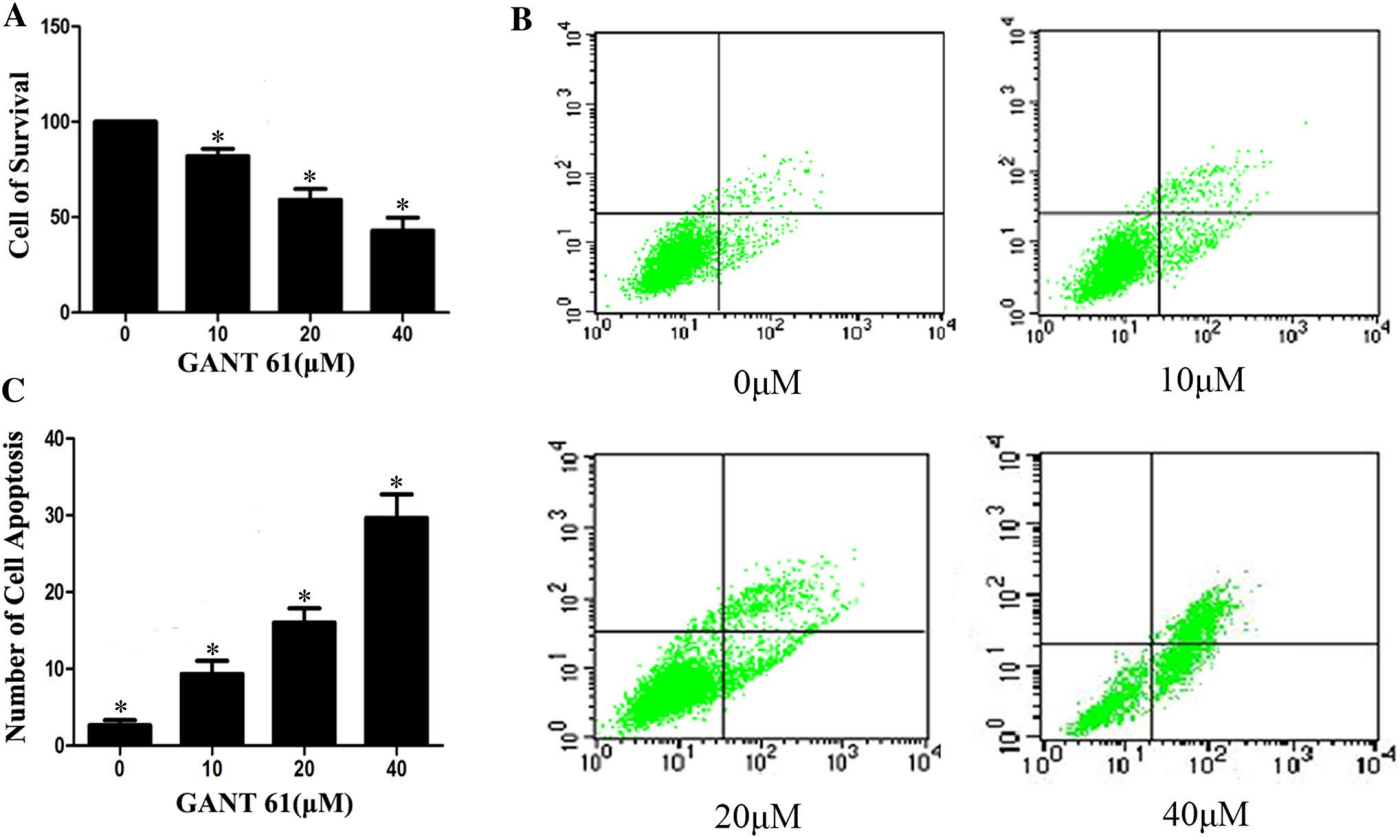
*GANT61 inhibits proliferation and promotes cell apoptosis of Daoy cells*. The effects of GANT61 treatment on proliferation were determined with CCK-8 assay. GANT61 inhibited the cell proliferation in Daoy cells (**a**). The inhibition was dose-dependent compared with the control group (*P* < 0.05). The effects of GANT61 treatment on cell apoptosis were determined with FITC-annexin V flow cytometry analysis. **b**, **c** The number of cells undergoing apoptosis increased significantly compared with the untreated group (*P* < 0.05)

To elucidate whether cell proliferation was decreased in the presence of GANT61, Daoy cells were treated with different concentrations of GANT61 for 24 h. Cell proliferation was determined with a CCK-8 assay. As shown in Fig. 2a, GANT61 significantly inhibited the proliferation of Daoy cells. This inhibition was dose-dependent and differed significantly from the control group (*P* < 0.05). To see whether GANT61 treatment could induce apoptosis, normally growing Daoy cells were treated with different concentrations of GANT61. After 24 h, the cells were subjected to flow cytometry. As shown in Fig. 2b, c, the number of cells undergoing apoptosis increased significantly compared with the untreated group (*P* < 0.05). These results verified the hypothesis that GANT61 induces apoptosis in Daoy cells.

### GANT61-induced alterations in the mitochondrial membrane potential promote apoptosis in Daoy cells

**Fig. 3:**
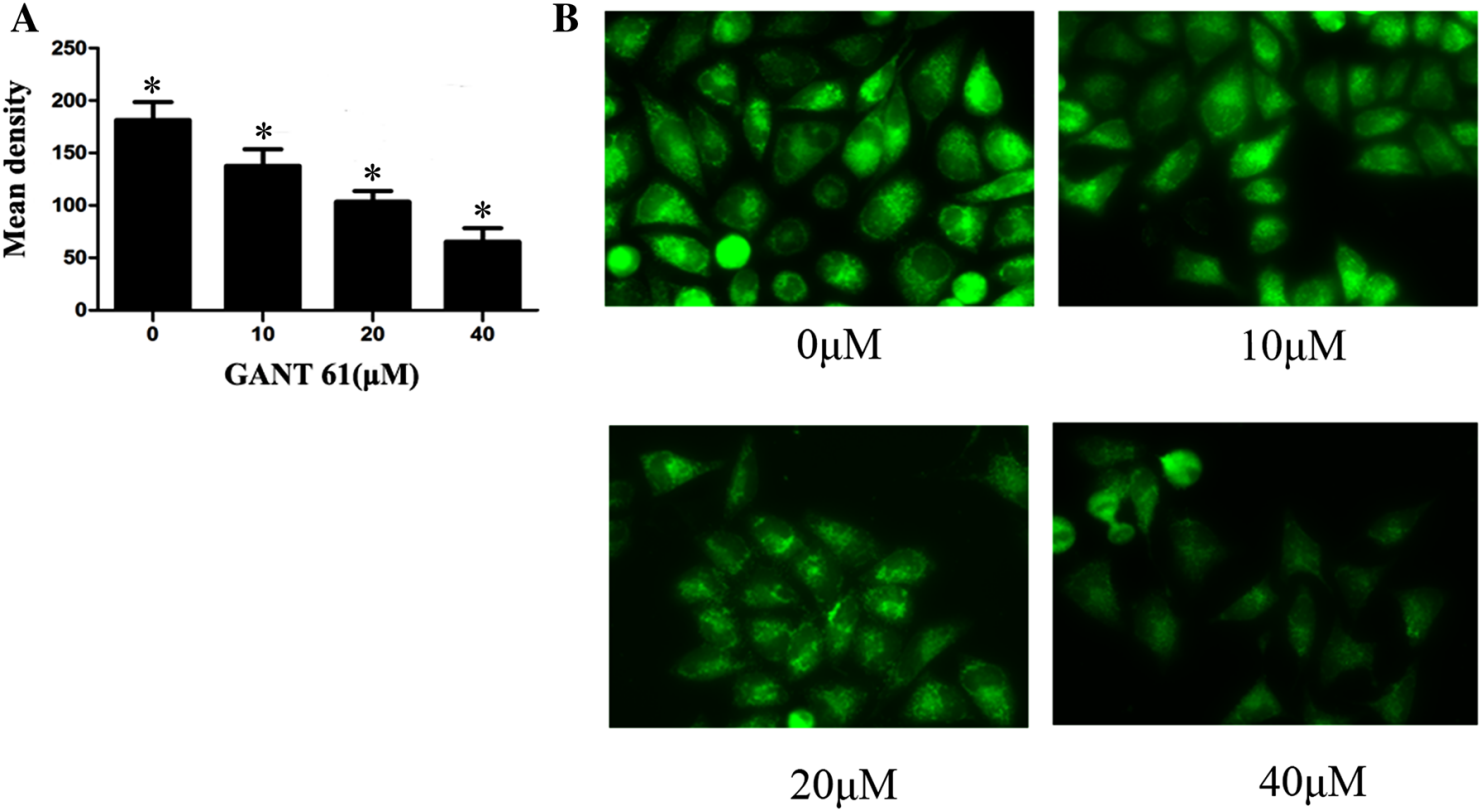
GANT61 inhibited mitochondria membrane potential promotes cell apoptosis of Daoy cells. The cells treated with different concentrations of GANT61 were subjected for rhodamine 123 after 24 h. **a**, **b** Mitochondria membrane potential decreased significantly compared with the untreated group (*P* < 0.05). The cells were mounted and observed under fluorescence microscope (×200)

To see whether GANT61 treatment could induce apoptosis through the mitochondrial pathway, normally growing Daoy cells were treated with different concentrations of GANT61. After 24 h, the cells were stained with rhodamine 123. As shown in Fig. 3a, b, the mitochondrial membrane potential decreased significantly compared with the untreated group (*P* < 0.05). These results verified the prediction that GANT61 induces apoptosis through the mitochondrial pathway.

### GANT61 inhibits the expression of GLI and Bcl-2 at both the mRNA and protein levels

**Fig. 4:**
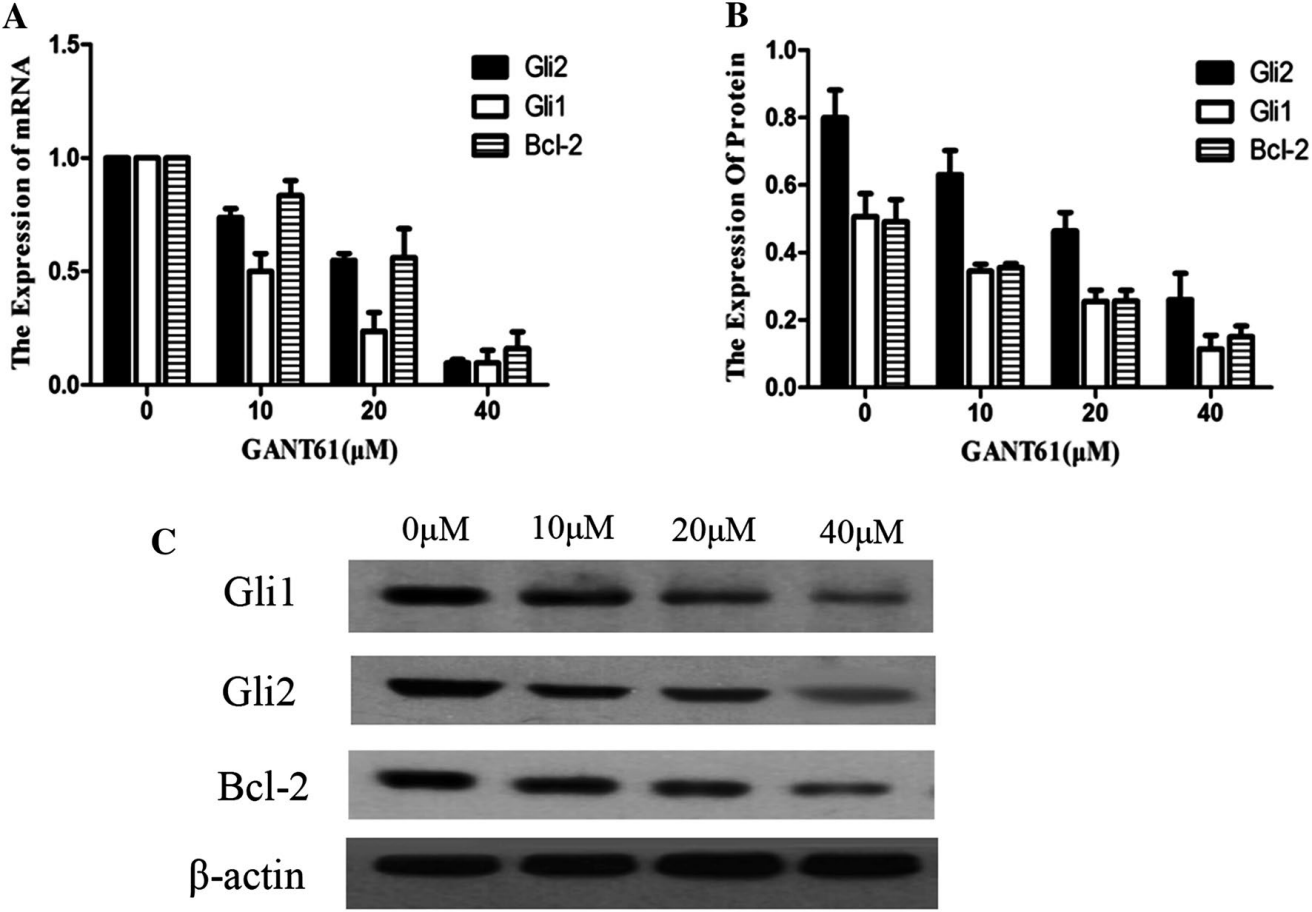
GANT61 inhibits the expression of GLI1, GLI2 and Bcl-2. **a** GANT61 significantly inhibited the expression of GLI1 and GLI2 (*P* < 0.05). Along with the decreased expression of the GLI1 and GLI2 genes, Bcl-2 mRNA appeared to be simultaneously down-regulated (*P* < 0.05). The effects of GANT61 treatment on the SHH signalling pathway at the protein level were assayed with Western blotting. **b**, **c** GANT61 can decrease the level of GLI1 and GLI2. With the decreased expression of the GLI protein, the Bcl-2 protein appeared to be down-regulated (*P* < 0.05). The inhibition of GLI and Bcl-2 by GANT61 occurred in a dose-dependent manner (*P* < 0.05)

To explore the mechanism linking cell apoptosis and GLI, total RNA was extracted from cells, reverse transcribed into cDNA and subjected to qPCR. GLI (GLI1 and GLI2) is an important transcription factor in the SHH signalling pathway, regulating the transcription of multiple downstream target genes. One of these targets is Bcl-2. Bcl-2 is an oncogene that controls cell apoptosis. As shown in Fig. 4a, GANT61 significantly inhibited the expression of GLI1 and GLI2 (*P* < 0.05). Along with the decreased expression of the GLI1 and GLI2 genes, Bcl-2 mRNA appeared to be simultaneously down-regulated (*P* < 0.05). The effects of GANT61 treatment on the SHH signalling pathway at the protein level were assayed with Western blotting. Figure 4b, c shows that GANT61 can decrease the level of GLI1 and GLI2. With the decreased expression of the GLI protein, the Bcl-2 protein appeared to be down-regulated (*P* < 0.05). The inhibition of GLI and Bcl-2 by GANT61 occurred in a dose-dependent manner (*P* < 0.05). These results are basically consistent with the qPCR data showing that GANT61 can significantly inhibit GLI1, GLI2 and Bcl-2 expression at the mRNA level.

### GANT61 affects the expression of Bax, caspase-3 and caspase-9, promoting cell intrinsic apoptosis

**Fig. 5:**
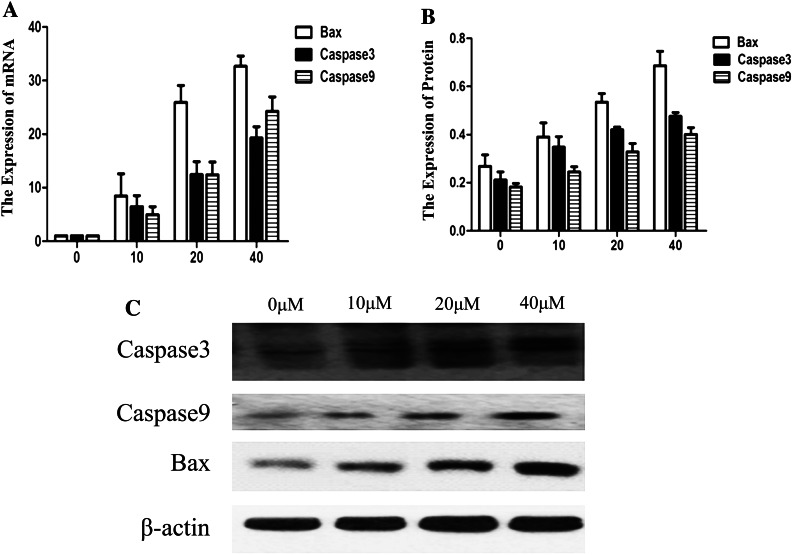
GANT61 maybe affects expression of Bax, caspase-3 and caspase-9 promoted cell intrinsic apoptosis. **a** Along with decrease in the expression of the GLI gene, GANT61 appears to simultaneously up-regulate Bax, caspase-3 and caspase-9 mRNA levels (*P* < 0.05). **b**, **c** Along with the decreased expression of GLI1, GLI2 and Bcl-2 protein, Bax, caspase-3 and caspase-9 protein appeared to be up-regulated synchronously

To further study the relationship between mitochondria and genes involved in cell intrinsic apoptosis, such as Bax, caspase-3 and caspase-9, the levels of these transcripts were detected through qPCR analysis. As shown in Fig. 5a, along with decrease in the expression of the GLI1 gene, GANT61 appears to simultaneously up-regulate Bax, caspase-3 and caspase-9 mRNA levels (*P* < 0.05). Figure 5b, c shows that, along with decrease in the expression of the GLI1, GLI2 and Bcl-2 proteins, GANT61 appears to simultaneously up-regulate Bax, caspase-3 and caspase-9 protein levels. These results show that GANT61 might have effects on mitochondrial-mediated cell intrinsic apoptosis.

### GANT61 enhances cisplatin-induced, caspase-mediated apoptosis in Daoy cells

**Fig. 6:**
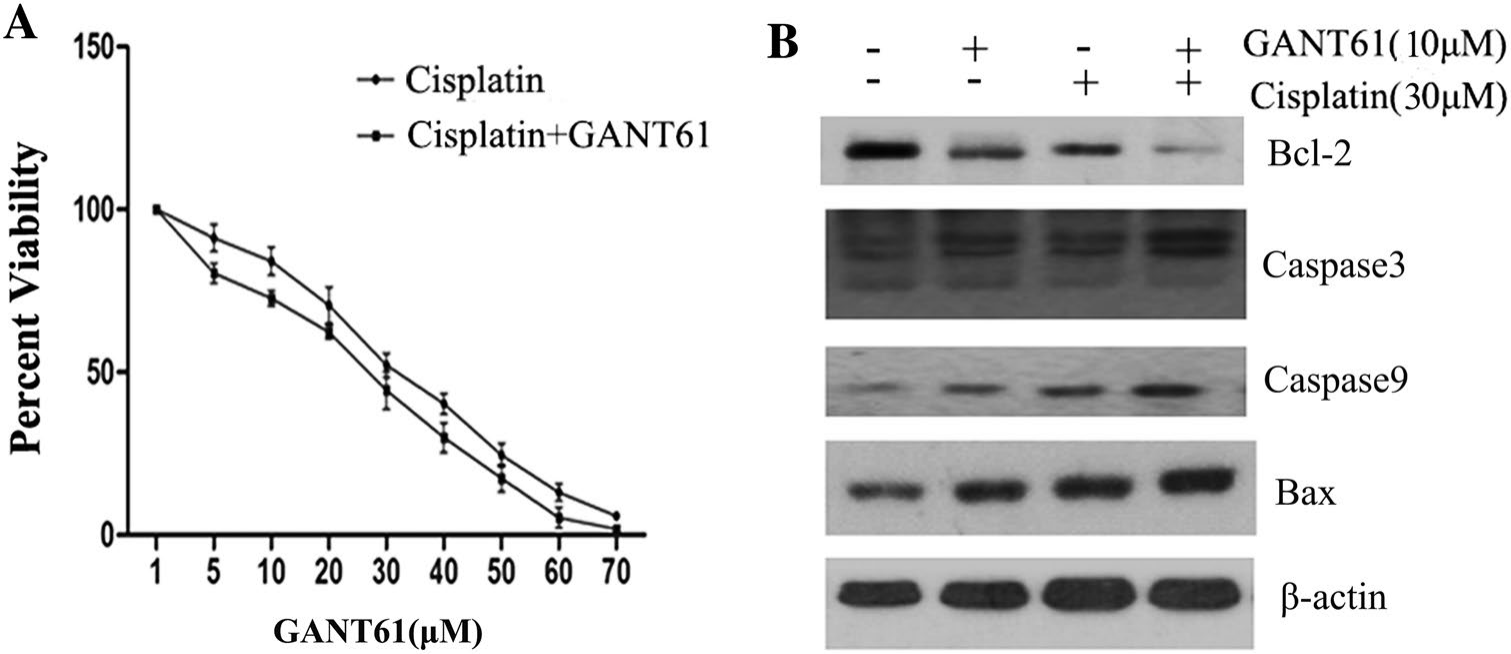
GANT61 enhances the antiproliferative effects of cisplatin. We further determined whether combining GANT61 with cisplatin would enhance the effect. **a** Combined treatment reduced the IC50 value of cisplatin from 34.47 to 30.02 µmol/L. **b** The down-regulation in Bcl-2 expression was significantly greater in the groups treated with both GANT61 (10 µm/L) and cisplatin (30 µm/L) than those in the single treatment group. However, combination treatment emerged greater increase in Bax, caspase-3 and caspase-9 protein expression than either drug alone group

The overexpression of GLI may be closely related to chemotherapy resistance, and the suppression of GLI can increase the sensitivity to chemotherapeutical drugs (Zahreddine et al. 2014). We further determined whether combining GANT61 with cisplatin would enhance the effect. Figure 6a show that combined treatment reduced the IC50 value of cisplatin from 34.47 to 30.02 µmol/L. To characterize GANT61-mediated enhancement of apoptosis, Daoy cells were exposed to different concentrations of GANT61 (10 µm/L) with or without cisplatin (30 µm/L) for 24 h. To investigate the pathomechanism leading to cell apoptosis, we investigated the combined effect of GANT61 and cisplatin on the expression of Bcl-2, Bax, caspase-3 and caspase-9. As shown in Fig. 6b, the down-regulation in Bcl-2 expression was significantly greater in the groups treated with both GANT61 (10 µm/L) and cisplatin (30 µm/L) than those in the single treatment group. However, combination treatment emerged greater increase in Bax, caspase-3 and caspase-9 protein expression than either drug alone group.

## Discussion

Medulloblastomas are the most common malignant brain tumours in children and are associated with significant morbidity and mortality. Several reports have shown that the SHH signalling system is highly activated in medulloblastoma (Justilien and Fields 2015; Matsumoto et al. 2014b). The zinc finger transcription factor GLI, including GLI1 and GLI2, is considered to be mediator of the SHH pathway in MB (Matsumoto et al. 2014b). To shed light on the role of GLI in MB, we screened for genes preferentially regulated by GLI. We showed here that the expression of the typical oncogene Bcl-2 is predominantly activated by GLI. To further clarify the mechanism of apoptosis, we investigated the relationship between Bax, caspase-3, caspase-9 and GLI.

GANT61 is one of the agents capable of inhibiting the SHH pathway. It acts by selectively binding to GLI and has also been found to suppress tumour proliferation. In our study, GANT61 inhibited tumour proliferation and induced cell apoptosis in vitro. Furthermore, we found that GANT61 can inhibit GLI, including GLI1 and GLI2, mRNA and protein expression. Bcl-2 is one of the oncogenes controlling apoptosis. We investigated whether GLI1 has a relationship with the typical oncogene Bcl-2 in cell apoptosis. Bcl-2 is a key protein in apoptosis and is highly expressed in multiple types of tumours (Janumyan et al. 2003). We found that the expression of GLI mRNA was significantly associated with Bcl-2 expression, as was protein levels. Suppressing the expression of GLI could inhibit the overexpression of Bcl-2 and the proliferation of tumour cells, simultaneously promoting apoptosis. Our findings suggest that Bcl-2 down-regulation may account for the increased cell death that occurs after the suppression of GLI in combination with DNA damage. Thus, it may be an attractive therapeutic strategy to block the expression of GLI in MB.

Bcl-2 plays an important role in the mitochondrial pathway of apoptosis (Wang et al. 2015). Bcl-2 can activate the transcription of a series of genes, such as Bax, caspase-3 and caspase-9 (Autret and Martin 2009). Caspases are crucial mediators of programmed cell death (Wen et al. 2012). Among them, caspase-3 is a frequently activated death protease and is essential for certain processes associated with apoptosis (Porter and Janicke 1999). Some pathways leading to caspase-3 activation have been determined to depend on caspase-9 function (Brentnall et al. 2013). Our results show that GANT61 can inhibit proliferation and promote cell apoptosis. Then, rhodamine 123 and flow cytometry showed that GANT61 might promote apoptosis by inhibiting the establishment of mitochondrial membrane potential. The qPCR and WB results show that GANT61 inhibits the expression of Bcl-2; however, Bax, caspase-3 and caspase-9 appeared to be simultaneously up-regulated. These results show that GANT61 might inhibit mitochondria membrane potential to promote cell apoptosis. Thus, blocking the expression of GLI might be an attractive therapeutic strategy for MB.

The overexpression of Bcl-2 is closely related to chemotherapy drug resistance, and the suppression of Bcl-2 has been shown to increase the sensitivity of tumour cells to chemotherapeutical drugs (Ogura et al. 2016). GANT61 can decrease Bcl-2 transcript levels through the down-regulation of GLI. We further assessed whether combining GANT61 with standard chemotherapeutic medicine would enhance this effect. The results showed that combined treatment reduced the IC50 value of cisplatin from 34.47 to 30.02 µmol/L. To investigate the role of the SHH signalling pathway in cisplatin resistance, we assessed the protein profile with Western blotting. GANT61 might activate Bax, caspase-3 and caspase-9 and inhibit GLI and Bcl-2. The enhancement in sensitivity to chemotherapeutical drugs is correlated with the suppression of Bcl-2 by GANT61.

Collectively, the SHH signalling pathway can regulate the tumour cell cycle and apoptosis at different molecular levels. The zinc finger transcription factor GLI plays an important role in the SHH signalling pathway. Our researches show that GANT61 promotes cell apoptosis and significantly inhibits cell proliferation and metastatic ability. GANT61 also can combine with cisplatin by enhancing cisplatin-induced apoptosis. Our findings show that GANT61 may be the effective targeted chemotherapeutic medicine in MB patients. Thus, it may be an attractive therapeutic strategy to block the expression of GLI in MB.

## Abbreviations

SHH: Sonic hedgehog
MB: Medulloblastoma
GLI: Glioma-associated oncogene homologue
Bcl-2: B cell leukaemia lymphoma-2
Caspase-3: Cysteinyl aspartate-specific proteinase-3
Caspase-9: Cysteinyl aspartate-specific proteinase-9
GANT61: GLI inhibitor
mRNA: Messenger ribonucleic acid
IC50: Half-maximal inhibitory concentration
DMSO: Dimethyl sulphoxide
